# ‘Beyond the Bump’: an online wellbeing and lifestyle pilot program during COVID-19 for first year postpartum mothers: a research article

**DOI:** 10.1186/s12884-022-04913-7

**Published:** 2022-07-25

**Authors:** Hannah E. Christie, Lauren A. Roach, Meredith Kennedy, Kassia Beetham, Barbara J. Meyer, Danielle Schoenaker, Monique Francois

**Affiliations:** 1 School of Medical, Indigenous and Health Sciences, The University of Wollongong, Wollongong, NSW Australia; 2 Illawarra Health and Medical Research Institute, Wollongong, NSW Australia; 3 Illawarra Diabetes Service, Illawarra and Shoalhaven Local Health District, Wollongong, NSW Australia; 4 School of Behavioural and Health Sciences, Australian Catholic University, Banyo, QLD Australia; 5 School of Primary Care, Population Sciences and Medical Education, Faculty of Medicine, University of Southampton, Southampton, UK; 6 NIHR Southampton Biomedical Research Centre, University of Southampton and University Hospital Southampton NHS Foundation Trust, Southampton, UK

**Keywords:** Postnatal, Exercise, Health, Mental Health, Community

## Abstract

**Background:**

Establishing a healthy lifestyle post-delivery is pivotal to reduce the incidence of chronic diseases. Due to COVID-19 restrictions, access to postpartum health programs has been increasingly difficult. The aim of this study was to inform, develop and evaluate Beyond the Bump (BtB); an online program to improve access to health and wellbeing education and support for physical activity in the postpartum.

**Methods:**

A three-phase mixed-methods design of a 10-week Australia-wide online pilot program during COVID-19 with women less than 1 year postpartum and their primary care health professionals was utilised. Phase-one: needs assessment focus groups and interviews. Phase-two: BtB program implementation pre-post health measures survey, attendance and engagement with the program. Phase-three: program evaluation with feedback surveys and interviews.

**Results:**

Women (*n* = 12) and health professionals (*n* = 16) expressed strong need for a postpartum program with access to education from experts on exercise, pelvic floor, sleep and baby nutrition. Despite BtB being developed from women’s suggestions (including time-of-day ‘morning’), attendance to all ten sessions was poor (of 162 registrations; 23% participated in the first session and 5% in the last session). Barriers to attendance included ‘too busy’,‘ forgot’ and ‘topic not relevant for age of child’. 88% of women reported the education as the most enjoyable component of the program. 100% (*n* = 26) of women interviewed would recommend the program to a friend.

**Conclusions:**

There is a continuing need for postpartum support. Online programs with access to expert education and exercise were reported to be of significant interest and value. However, more research is needed to improve the uptake and value placed on mothers’ wellbeing and physical activity.

**Supplementary Information:**

The online version contains supplementary material available at 10.1186/s12884-022-04913-7.

## Background

The postpartum period is a significant time in a woman’s life, with many emotional and physical changes. The physical, emotional and social support for the wellbeing of mothers is essential in this transition. To reduce the risk of chronic diseases such as obesity, diabetes and cardiovascular disease there is an emphasis on returning to a healthy weight in the postpartum [1]. Improving health behaviours during the postpartum period will support women’s health and wellbeing [2]. Whilst there are many programs to support health during pregnancy, few programs exist in the postpartum that support and encourage women to focus on their wellbeing and continue a healthy lifestyle of nutrition and physical activity.

Physical activity is cornerstone for women’s health and wellbeing during the postpartum and intrapartum periods. Physical activity lowers the risk of mental health conditions and co-morbidities such as obesity, type 2 diabetes and cardiovascular disease [3–6]. However, only half of postpartum women in Australia reported receiving physical activity education, and only 63% meet the physical activity recommendations in the postpartum period [7].

Postpartum programs that include education and support for wellbeing [4], physical activity and nutrition [5] have been shown to improve subsequent outcomes. For example, women who participated in a two-month once weekly in-person education and physical activity program reported decreased mental health symptoms, compared to women who received written education only [4]. A separate study has also demonstrated, an intensive four-month program of physical activity and nutrition classes 3–4 times per week increased physical activity levels, but not nutritional adequacy, in sedentary postpartum women [5]. These programs show that by providing group education on a broad range of wellbeing topics, with and the inclusion of physical activity sessions can improve postpartum lifestyle and wellbeing. However, the lack of such programs may be a barrier to women achieving a healthy lifestyle in the postpartum period.

The transition to motherhood is known to present more and new barriers to completing the traditional structured physical activity recommendations [8, 9] which has been further exacerbated by restrictions during the COVID-19 pandemic. Women reported having received subpar care during their pregnancy, delivery, and postpartum period due to COVID-19 disruptions to health services, further, these women reported that of the support they had received, they were unable to source what they were looking for [10, 11]. A recent survey of postpartum women during COVID-19 highlighted the need for a community program targeted at providing education and physical activity [12]. Expert monitored web-based platforms provide an important alternative for overcoming barriers such as lack of time [8] and not prioritising health of mother [9]. Studies have found women with access to web-health services presented improved self-efficacy and reduced postnatal depression compared to standard in-person care [11]. Therefore, the potential for web-health to support more women and provide access to experts from home is promising.

The aim of this pilot feasibility study was to identify barriers, and inform, develop and evaluate the Beyond the Bump (BtB) postpartum program to improve access to expert education on health and wellbeing, and support women to be more physically active in their first year postpartum.

## Material and methods

**Fig. 1:**
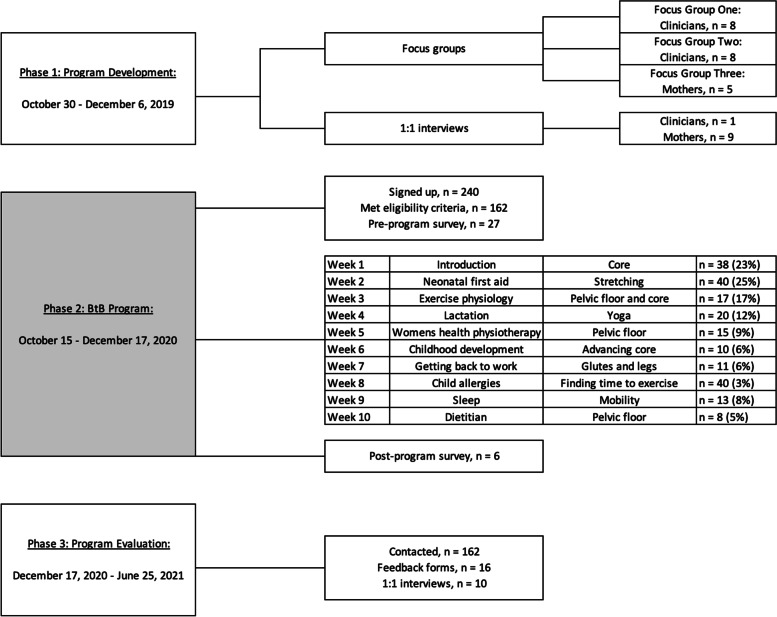
Schematic of BtB program development, implementation, and evaluation

Study design: A three-phase study design was used to inform, develop, and evaluate ‘Beyond the Bump’ (BtB) an online postpartum program (Fig. 1). BtB was originally intended to be in-person; however, due to COVID-19 restrictions an online webinar platform was utilised. Phase one of the study involved a needs assessment conducted using focus groups with women less than 1-year postpartum and health professionals. Phase two of the study involved developing and implementing a BtB program based on needs identified in phase one (comprising of ten weekly, live education and physical activity sessions). Phase three of the study involved an evaluation of BtB with interviews of women who registered for the program. Recruitment and ethics: Participants for phases one and two were recruited via Australia-wide social media advertisement and referrals from health professionals. Health professionals (including dietitians, physiotherapists and nurse educators) were recruited for phase one needs assessment via word of mouth and email. Women who registered and participated in phase two BtB program were then invited to participate in phase three evaluation. This study was approved by the University of Wollongong Human Ethics Committee (HREC: 2019/ETH13571). Informed consent was provided by all women.

### Phase one: needs assessment

#### Study design and protocol

To design BtB, semi-structured face-to-face, focus-groups, and telephone interviews were conducted with women less than 1-year postpartum and relevant health professionals. Questions and discussion points were developed with input from researchers, postpartum women and health professionals. These included discussion points on the format, education topics and locations/time-of-day of the proposed postpartum program. Focus groups and interviews were audio recorded, transcribed verbatim, and verified with participants de-identified. Two researchers (HEC and LAR) decided on data saturation once similar ideas about the logistics of the program were repeated.

#### Data analysis

Two researchers (HEC and LAR) independently coded typed transcripts, NVivo [QSR International Pty Ltd. (2020). NVivo (released March 2020)(Computer Software)] was used to organise this data. Investigators used a Framework Analysis Method [13] to analyse data. A thematic framework was utilised and transcripts were coded separately. Next, direct quotes and themes were indexed to determine the richness of themes. Subthemes were subsequently determined. Lastly, key themes and direct quotes were tabulated (S1).

### Phase two: Beyond the Bump development and implementation

#### Study design

The above needs assessment was used to develop and implement BtB. BtB included ten, weekly live education and physical activity sessions online (Zoom video communications Inc., 2016) (Fig. 1). Each session had 20-min education, 10-min question and answer (Q&A), and 20-min of physical activity. Women asked questions during the sessions using the chat function, monitored by a researcher. Registration occurred via Eventbrite (Eventbrite, 2020; www.eventbrite.com.au/e/122499716951), attendance was recorded at each session and women who registered were invited to fill out an online survey [Qualtrics (2005) (Version December 2020) (Website)] to assess overall health and wellbeing pre and post program. Eligibility criteria for the research component included: aged above 18 years, women less than 1-year postpartum, and access to internet connection. A closed Facebook group was also created with attendees.

#### Health measures survey

Demographics including age, months since delivery, education, and postcode were collected, followed by questions/validated surveys on wellbeing, mental health, and health related behaviours.

Mental health symptoms were measured with the *Depression, Anxiety, and Stress Scale–21 items (DASS) * [14] (Cronbach’s alpha for depression, anxiety, and stess are 0.94, 0.87, 0.91 respectively [12]). Wellbeing/Health-Related Quality of Life was assessed using the *36-item Short Form Health Survey questionnaire (SF-36) * [15]*(*Cronbach’s alpha for physical functioning: 0.93, social functioning: 0.73; physical role limitations: 0.96; emotional role limitations: 0.96; bodily pain: 0.85, mental health: 0.95; energy/vitality: 0.96; and general health: 0.95 [16]). Sleep was assessed using a 4-item questionnaire evaluating the length of sleep, the length of uninterrupted sleep, and awake hours during night (all measured in hours). In addition, interruptions were assessed on a categorical scale of 0–1, 2–3, 4–5, 6–7, and 7 or more times sleep was interrupted per night. Perceived value of outcomes for general health was assessed using a 12-item outcome expectations measure designed to be relevant to the study population (Table 1)*.* Each item was scored on a 9-point Likert scale ranging from 1 (no value) to 9 (the highest of value) [17]. Physical activity was assessed using the validated *Godin-Shephard Leisure-Time Physical activity Questionnaire* (Godin Score; kappa index 0.74) [18]. Physical activity minutes per week were then calculated from the reported Godin scores. *Dietary frequency of consumption* of the core food groups (including vegetables [fresh], vegetables [frozen], fruit (fresh), fruit [frozen], grains, legumes, meat, dairy, and snacks) were assessed using a questionnaire developed in line with Australian Dietary Guidelines [19].

**Table 1:** Pre- and post-program (10 weeks) participant characteristics and health behaviours and values

	Pre-program *n* = 6	Post program *n* = 6	*P* value Pre v post
BMI (kg.m^−2^)	22.1 ± 0.30	22.7 ± 1.87	0.599
Physical Activity (min.wk^−1^)	135 ± 53	123 ± 59	0.566
Godin Score (a.u.)	37.80 ± 14.48	35.00 ± 14.20	0.661
Strenuous	1.00 ± 0.00	1.00 ± 0.00	1.000
Moderate	2.40 ± 2.19	2.20 ± 1.30	0.861
Light	5.60 ± 2.07	5.00 ± 2.92	0.208
*Sleep (n)*			
Total sleep (hours)			0.513
0–1	0	0	
2–3	0	0	
4–5	0	0	
6–7	2	3	
7 +	2	1	
Uninterrupted sleep (n)			0.572
0–1	0	0	
2–3	2	2	
4–5	0	1	
6–7	2	1	
7 +	1	0	
How many times sleep interrupted? (n)			0.287
0–1	1	1	
2–3	3	0	
4–5	0	1	
6–7	1	0	
7 +	0	2	
Awake time during night (hours)			0.721
0–1	2	1	
2–3	1	1	
4–5	1	2	
6–7	0	0	
7 +	1	0	
*Diet (n)*			
Vegetables (Fresh)			1.000
Daily	4	4	
Most days a week	1	0	
1–2 times a week	0	0	
Rarely	0	0	
Vegetables (Frozen, dried, or canned)			0.465
Daily	0	0	
Most days a week	0	0	
1–2 times a week	2	0	
Rarely	3	4	
Fruit (Fresh)			0.549
Daily	3	4	
Most days a week	1	0	
1–2 times a week	1	0	
Rarely	0	0	
Fruit (Frozen, dried, or canned)			1.000
Daily	1	0	
Most days a week	1	1	
1–2 times a week	0	0	
Rarely	3	3	
Grains			0.513
Daily	3	3	
Most days a week	2	1	
1–2 times a week	0	0	
Rarely	0	0	
Meat			0.285
Daily	4	4	
Most days a week	1	0	
1–2 times a week	0	0	
Rarely	0	0	
Legumes			0.082
Daily	1	2	
Most days a week	0	1	
1–2 times a week	3	1	
Rarely	0	0	
Dairy			0.549
Daily	3	1	
Most days a week	1	0	
1–2 times a week	0	3	
Rarely	0	0	
Snacks			0.549
Daily	1	1	
Most days a week	0	0	
1–2 times a week	1	0	
Rarely	1	2	
*DASS (a.u.)*			
Depression	2.80 ± 3.03	2.50 ± 3.79	0.391
Anxiety	2.60 ± 3.44	3.75 ± 3.86	0.391
Stress	5.40 ± 3.51	6.50 ± 4.43	0.444
*QoL (SF-36) (a.u.)*			
Physical Functioning	92 ± 15	90 ± 14	1.00
Role physical	80 ± 45	63 ± 43	0.752
Role emotional	87 ± 18	58 ± 50	0.252
Energy/fatigue	48 ± 20	40 ± 19	0.391
Emotional wellbeing	80 ± 14	81 ± 21	0.854
Social functioning	73 ± 22	78 ± 12	0.391
Bodily pain	83 ± 23	84 ± 0.44	0.444
General health	74 ± 4.2	73 ± 13	0.861
*Values (a.u.)*			
Socialise with friends	7.60 ± 1.14	7.50 ± 1.73	0.604
Get myself out of the house	8.00 ± 1.41	7.00 ± 1.83	0.444
Meet new people	4.40 ± 2.07	3.75 ± 3.20	0.547
Praise from friends and family for being physically active	4.80 ± 2.59	4.25 ± 3.77	0.547
Weight control	5.60 ± 2.88	6.75 ± 2.06	0.213
Increase fitness	7.20 ± 1.64	7.25 ± 2.06	0.391
Lower the risk of type 2 diabetes	6.80 ± 3.35	6.75 ± 3.86	0.718
To have more energy	8.00 ± 1.73	6.25 ± 3.59	0.133
Make me feel better physically	8.00 ± 1.73	8.00 ± 1.41	0.638
Reduce stress	7.60 ± 2.19	6.33 ± 4.62	0.423
Better overall mood	8.20 ± 1.79	7.00 ± 4.00	0.391
Sense of accomplishment	7.60 ± 1.67	8.00 ± 2.00	0.391

Values are reported in mean ± standard deviation or number of participants (n). Number of participants vary due to missing data. Dependent t-tests and Chi-squares determined *p* value

#### Data analysis

Dependent t-tests (continuous variables) and Chi-square tests (categorical variables) were then used to compare pre and post program health outcomes. Statistical significance was noted as *p* < 0.05.

### Phase three: evaluation of Beyond the Bump

#### Study design

Participants who registered for BtB (phase two) were invited to complete an online survey and phone interview around their participation in the program (or lack thereof), as well as any further feedback they could offer. Women who completed the survey went into a draw to win one of two $100 grocery gift cards.

#### Measures

An online survey [Qualtrics (2005) (Version February 2021) (Website)] was sent to all women to ask for feedback on the program including questions such as: ‘what did you like about BtB’, ‘what did you dislike about BtB’, and reasons for attending or not attending the program.

Interviews were then conducted in a semi-structured context. Conversations were audio recorded, transcribed verbatim, and accuracy verified. All participants were de-identified. Two researchers (HEC and LAR) decided on data saturation once participants began repeating similar themes.

#### Data analysis

Two researchers (HEC and LAR) independently coded typed transcripts, NVivo [QSR International Pty Ltd. (2020). NVivo (released March 2020)(Computer Software)] was used to organise this data. Investigators used a Framework Analysis Method [13] to analyse data. A thematic framework was utilised and transcripts were coded separately. Next, direct quotes and themes were indexed to determine the richness of themes. Subthemes were subsequently determined. Lastly, key themes and direct quotes were tabulated (S1).

## Results

### Phase one: needs assessment

Twelve postpartum women and 16 health professionals participated in individual interviews and two focus groups (FG). Five major themes emerged: time/day, location, format of events, topics, and barriers. Direct quotes are listed in S1. Data for this phase was collected prior to COVID-19.

#### Time and day

All participants agreed sessions would be best held in the morning after ‘*dropping off other kids to school’* (FG1) as ‘*anything in the afternoon seems to mess with their sleep and evening routine’* (P1]) and that ‘*timings change as the baby grows’* (P14). However, the day to have the program was dependent on the working status of the individual, for example, whilst most women agreed *‘weekends everybody is with their family’* (P14) one woman explained ‘*I’ll be back at work … so Friday to Sunday is good’* (P5). Health professionals expressed concern about attendance for women returning to work and to avoid school holiday periods.

#### Location

It was quickly agreed ‘*women are unwilling to travel particularly far*’ (P7) and will ‘*come to the one that is close to them*’ (P10). Participants discussed first time mothers may prefer different locations to women with more than one child. For example, ‘*if it is first time mothers then like an inside place’* (P9) and ‘*anyone with older children … it’s always good to be near a playground’* (P13). Health professional focus groups and interviews felt locations should not change each week as participants would be more inclined to attend if they knew where they were going and could build the program easily into their weekly routine. Similarly, there was much discussion around potential wet weather locations with suggestions including the ‘*university aquatic recreation centre’ (*FG1)*, ‘surf clubs’* (FG1)*, ‘local shopping centres* (FG1)*’,* and finding a space that is *‘pram friendly … plenty of space for paths, parking, those sort of things’* (Health professional 1).

#### Format

Women agreed education should go at the start of each session followed by physical activity (had planned on a walk when face-face) as then women can talk to health professionals and *‘other mums on the walk about stuff you’ve learnt or share experiences’* (P8). Health professionals raised concerns of how sessions would work, referral pathways and the potential for anonymous question and answer periods. One health professional also discussed the potential for having ‘*the [education] first so that then, they’ve got the opportunity to ask questions privately’* (FG2)*.*

#### Topics

Topics women suggested are divided into subthemes of mother’s health and baby’s health. Topics for mother’s health included, *‘exercise for preventing diabetes type 2 in the future as well as GDM [gestational diabetes] in the next pregnancy’* (P5), *‘Pelvic floor physio’* (P7), *‘good exercises postpartum … at different stages’* (P9)*, ‘maybe like a postpartum psychologist’ (P12), and ‘how your intimate relationship with your partner changes’* (P14). Topics for the baby’s health included, *‘a GP [general practitioner] or something on childhood illnesses or rashes and things to look out for’* (P7), *‘sleep’* (P8)*, ‘pediatrics dietitians’* (P12)*, ‘psychologist regards to my older child and how he’s coping with the transition of having a sibling* (P13), *and ‘a baby nutritionist … But also, first aid would be amazing’* (P14).

#### Barriers

Potential barriers to participation included being on time to anything *‘I wouldn’t start the education for 15 min after it’s meant to start or something’* (P7). Questions were raised by women about education groups based on child’s age as the first-year presents vast differences in development and information relevance as *‘if there were a group of women who started and everyone in the group had a baby between, I don’t know, who was born in a month or two months, then we’d all have similar issues’* (P14). These barriers were similarly discussed in health professional focus groups, and also included time *‘I’m never on time for anything now’* (FG1), the chaotic nature of parenthood *‘life, is so chaotic when you have a baby, you know, just getting out of the house on time’* (FG1*)* and participants being already overburdened with appointments *‘The majority of women they’re still working, they’ve got other little kids and … it’s just so many appointments’* (FG1).

### Phase two: BtB program implementation

#### Attendance to BtB

Two hundred forty participants registered for BtB and 162 of these women met eligibility criteria (most were ineligible due to currently being pregnant, or more than 1-year postpartum). Of those eligible, mean attendance was 11.2%. Attendance decreased substantially as the program continued (attendance for each session provided in Table 2). Of the 162 eligible women, only four attended most of the (8–10) sessions. The program was run on a Thursday morning at 9:30am as decided by the needs assessment.

**Table 2:** Feedback for Beyond the Bump as given by women who attended the program

	N	%
Most liked		
Education	14	86
Activity	3	19
Opportunity to ask questions	5	31
Other	0	0
Most disliked		
Program length	2	13
Program time	5	25
Topics were not relevant	1	6
Other	5	31
Sessions attended		
0	0	0
1–4	10	63
5–7	5	31
8–10	2	13
Reasons for not attending		
Too busy	6	38
Not interested	1	6
Not relevant	4	25
Other	1	6
Recommend to a friend?		
Yes	16	100
No	0	0

#### Engagement

Participants were encouraged to interact during the sessions with the chat function. Strong engagement was made in every session of the program. Examples included comments such as: ‘*I’m glad that I watched this before hitting the gym to realise that I needed to start back really slow with foundational exercises since our bodies change so much during pregnancy’* and ‘*I just wanted to thank you for putting this together. I’m really excited. I’m a mum of twins, 10 weeks old. One twin has been home for 5 days, the other is still in special care. It’s great this session is online so I can dial in as I’m on the move between home and the hospital. It would be great if you could talk more about physio health. I went and saw one recently and really benefitted from it.’*

#### Health outcomes survey

Twenty-seven women (33 ± 4 years, BMI 24 ± 6 kg.m^2^, 1.0 ± 0.1 children) completed the pre-program survey, however, only six also completed the post-program survey. Of these six women the mean age was 34.6 ± 2.3 years, four had a bachelor (or higher) degree, and were an average of 6.0 ± 4.2 months postpartum (youngest child). Maternal physical activity, nutritional intake, sleep, wellness, mental health, or values to health after the program were not different (all *p* > 0.05, Table 1). Women who did not complete the post-program survey had a significantly higher BMI (23.4 ± 9.2 v 22.1 ± 0.3 kg.m^2^; *p* = 0.032) and carried out more strenuous physical activity (1.5 ± 1.15 v 1.00 ± 0.00 a.u.; *p* = 0.026) compared to women who did complete the post-program survey.

### Phase three: evaluation

Twenty-six women participated in phase three: *n* = 10 one-on-one interviews, and *n* = 16 feedback forms. Seven major themes emerged in interviews including: social media, activities/topics, attendance/barriers to attendance, recommend to a friend, structure, timing, and platform. Direct quotes are listed in S1.

#### Feedback form

Sixteen women filled out the feedback survey. The most liked part about the program was the education (88% liked), interestingly the most disliked part of the program was the time-of-day (25% disliked). It was reported over half of women (63%) attended 1–4 sessions, and top reasons for not attending included being too busy (38%) and the program not being relevant (25%) (Table 2). 100% of women agreed they would recommend BtB to a friend.

#### Interviews

##### Social media

Most participants heard about BtB from Facebook advertising, and some participants registered after friend’s referral ‘*it’s actually really good’* (P19). Most women joined the closed Facebook group, which offered recording of videos however they felt differently about navigating it: for example, they *‘didn’t feel it was easy [to navigate]’* (P16) or ‘*it was fine to just scroll back down’* (P22). The Facebook group also offered opportunities to interact with experts and other mothers though ‘*felt that [having participants be interactive on the Facebook page] wasn’t really happening’* (P16) but ‘*I thought it was a really good idea’* (P21).

##### Activities or topics

Women found a handful of topics helpful during the sessions. These sessions included: ‘*women’s health physio’* (P16), ‘*the working mums one’* (P8), and ‘*the session on breastfeeding’* (P21). Other mothers commented that ‘*it was more irrelevant for me’* as *‘my little guy … was already past six months and being a second time mum’* (P18). Women also mentioned the lack of interest in the exercise portion of all the sessions.

Interestingly, when mentioning potential topics for another program, women mentioned those in this series. For example, women often mentioned ‘*planning to prepare for work’* (Participant 18), ‘*one on sleep’* (P21), and ‘*someone talking about language development’* (P16).

##### Attendance/Barriers to attendance

Reported barriers to attendance was consistent. Women reported ‘*remembering was a problem’* (P15) and ‘*if it didn’t fit in with their [children’s] schedule then I just wouldn’t do it’* (P15). Other reasons included not being able to ‘*hear it over the screaming’* (P19), and ‘*being released from a 4-month lockdown and sitting behind a screen … was the last thing I ever felt like doing’* (P23).

##### Recommend to a friend?

All women agreed they would recommend the program to a friend, and that they could ‘*definitely see the value of it though if I was starting fresh’* (P18).

#####  Structure

Women commented the structure of the program was a ‘non-problem’ (P18) and felt the ‘*Q&A is always great as well’* (P20), however ‘*the potential to submit pre-questions’* (P18) may be of value.

##### Timing

Optimal day and time suggestions for the program varied. Whilst some mothers ‘*were free’* (P15), others found it difficult with ‘*the kids needing to go to sleep’* (P21) or ‘*having two other children’* (P24). Other ideas included, ‘*not having the exact same time for every session’* (P21) or *‘evening times’* (P21).

##### Platform

Mixed feedback was given for the COVID-adjusted webinar platform. Many agreed ‘*webinar was the best way because of covid’* (P19) and ‘*it was just so good to do from home’* (P22). Many also suggested ‘*audio is definitely easier’* (P15) as they ‘*don’t really have the time to sit down and watch videos’* (P17). Interestingly, others suggested ‘*would have liked an excuse to go to something’* (P21) in-person and to have ‘*met other mums in that situation’*, however were sympathetic to this platform ‘*because of the times we live in now’* (P22).

## Discussion

Continual growing need exists for accessible programs that support postpartum health and wellbeing [10, 11, 20]. Beyond the Bump (BtB), which involved online weekly education and physical activity, was developed to improve access to postpartum support and empower women to improve their health and physical activity. Despite significant interest and reported need for the program, attendance was poor; 23% attended the first session and 5% the 10^th^ session. Both health professionals and postpartum mothers interviewed expressed need for education on postpartum exercise, pelvic floor, returning to work and sleep. Women who attended the live webinar sessions showed consistently high engagement with health professionals, suggesting women valued gaining information from live experts. Upon program evaluation, women who didn’t attend reported the program included topics were not always relevant to their postpartum stage, and that the live webinar format led to difficulties in time attending with the unpredictable nature of their day.

Barriers to attendance in programs developed for postpartum mothers are widely reported [8, 9]. Of interest, the barriers mentioned in the pre-program needs assessment (getting out of the house on time and baby’s unpredictable routine) were the same reasons women gave for not attending BtB. Here, despite utilising both live and recorded sessions women reported they were unlikely to watch later. Previous research reports similar issues with physical activity postpartum in programs similar to ours. ‘Mums on the Move’ [21] attempted to mitigate maternal barriers of child-care and time management by providing a free bike or treadmill for at home sessions. However, there was no increase in postpartum physical activity levels. In contrast, Norway’s Fit For Delivery trial [22], which provided women with a free exercise program during their pregnancy had high adherence and compliance to physical activity sessions. This may be due to mother’s placing higher value on the health of their child whilst pregnant, and once the baby is born, the importance is less on them and more on the baby [8, 9]. Whilst an ‘intention to change questionnaire’ was not carried out with women, their registration for the program suggests they were interested in learning more about healthy behaviours, though due to low attendance rates, it suggests women were not able to prioritise the program. Mobilising a program during pregnancy with a clear alignment and pathway into the postpartum may improve attendance and physical activity postpartum.

Program topics of ‘pelvic floor physio’, ‘sleep’, ‘paediatric dietitians’ and ‘first aid’ were reported to be of high value to women. Providing these topics live by experts was also considered important by health professionals, giving an opportunity to receive accurate, evidence-based advice and ask questions directly. Whilst 100% of women reported they would recommend the program to a friend, many did not regularly attend sessions. Studies have reported women want, and react positively to, social support, examples of a positive motherhood experience, and regaining health and wellbeing for their baby and themselves [20, 23]. Whilst support from partners, mothers, and friends is of primary need, support from other mothers is also sought-after [20]. Decreasing program attendance may be due to Zoom fatigue over the last 2 years of COVID-19 lockdown and the lack of use of the private support group may be attributed to fear of judgement, criticism, and wanting independence [20]. Unfortunately, due to COVID-19 we were not able to run the in-person program, however given the reported barriers it is unclear if this would have been more successful.

Though physical activity is imperative for health in postpartum and intrapartum periods, participation remains low. Health practitioners agreed exercise would be beneficial, in line with women expressing their need for education on appropriate exercises. Each session included 20-min of physical activity led by a postpartum expert. However, upon feedback, woman reported they weren’t interested in the physical activity. Whilst we considered the importance of postpartum physical activity a primary take home message and the physical activity sessions provided online were easy to follow, this was not enough to engage women, as many dropped off during this session. Previous studies suggest varied opinions on whether in-person or online programs/exercise sessions may provide women with accountability to participate in physical activity and accommodate for women’s competing priorities [3, 24–26]. Given the current climate of continuing disruptions and uncertainty, future research should continue to explore virtual, physical activity programs that are engaging and hold women accountable to lead to longer-term behavioural changes.

### Strengths and limitations

This study comprehensively reports on the development and evaluation of an online physical activity and wellbeing postpartum program. A strength was providing women with a needed access to health professionals [10, 11] and adapting to COVID-19 with a virtual format; potentially allowing more women in regional areas access to experts. The inclusion of postpartum mothers and health professionals in program design was a strength as the content and elements were based on evidence and needs, however, using the same women in all three phases would provide further context to program attendance outcomes. The sample sizes for the surveys are a limitation, despite having incentives (grocery voucher draw) to complete surveys only a handful of women did so. Based on feedback surrounding time management, future research could investigate smaller ‘bite size’ sessions to reduce the time commitment. Similarly, the program’s decreasing attendance was unexpected and may be due to online platform fatigue due to the timing of this study after COVID-19 lockdowns. In addition, future studies may find attendance improved by the provision of a non-live program women can access anytime across a week. Additionally, future research should attempt to bridge the intention-behaviour gap by creating a program that extends from pre-conception, during and following pregnancy allowing to continual ongoing support.

## Conclusion

Physical activity and postpartum education are essential to improve wellbeing and reduce the risk of illness/chronic disease. The postpartum period has significant gaps in support provided to women, especially for physical activity. The BtB program was developed alongside health providers and postpartum mothers to support and encourage health behaviours. Whilst the program met the needs analysis in phase one, attendance to the program, and therefore changes in health behaviours were minimal. Future postpartum programs may benefit from integrating other platforms of use and separate the program by months postpartum and relevant information needed during this time.

## Supplementary Information

**Additional file 1: Supplementary Table 1. **Identified themes for needs analysis and evaluation of a postpartum community program.

## Data Availability

The datasets used and/or analysed during the current study are available from the corresponding author on reasonable request.

## Abbreviations

BMI: Body Mass Index
BtB: Beyond the Bump
COVID-19: Coronavirus
DASS: Depression Anxiety Stress Scale
FG: Focus Group
Godin: Godin-Shephard Leisure Time Physical Activity Questionnaire
ISLHD: Illawarra Shoalhaven Local Health District
Q&A: Questions and Answers
RAND: Research and Development
SF-36: Short Form Health Survey Questionnaire
