# Correction: ‘Beyond the bump’: an online wellbeing and lifestyle pilot program during COVID-19 for first year postpartum mothers: a research article

**DOI:** 10.1186/s12884-022-05068-1

**Published:** 2022-10-07

**Authors:** Hannah E. Christie, Lauren A. Roach, Meredith Kennedy, Kassia Beetham, Barbara J. Meyer, Danielle Schoenaker, Monique Francois

**Affiliations:** 1 School of Medical, Indigenous and Health Sciences, The University of Wollongong, Wollongong, NSW Australia; 2 Illawarra Health and Medical Research Institute, Wollongong, NSW Australia; 3 Illawarra Diabetes Service, Illawarra and Shoalhaven Local Health District, Wollongong, NSW Australia; 4 School of Behavioural and Health Sciences, Australian Catholic University, Banyo, QLD Australia; 5 School of Primary Care, Population Sciences and Medical Education, Faculty of Medicine, University of Southampton, Southampton, UK; 6 NIHR Southampton Biomedical Research Centre, University of Southampton and University Hospital Southampton NHS Foundation Trust, Southampton, UK

**Correction: BMC Pregnancy Childbirth 22, 591 (2022)** 10.1186/s12884-022-04913-7

Following publication of the original article [1], the author reported that authors Hannah Christie, Lauren Roach, Barbara Meyer and Monique Francois should be affiliated to:

1. School of Medical, Indigenous and Health Sciences, The University of Wollongong, Wollongong, NSW, Australia.

2. Illawarra Health and Medical Research Institute, Wollongong, NSW, Australia.

This have been amended to the author group and are presented correctly in this correction article.

The original article [1] has been corrected.
