# Supplementary material for: ‘Beyond the Bump’: an online wellbeing and lifestyle pilot program during COVID-19 for first year postpartum mothers: a research article
Source: BMC Pregnancy Childbirth. 2022 Jul 25;22:591. doi: 10.1186/s12884-022-04913-7 (PMC9310358; doi:10.1186/s12884-022-04913-7)
Supplement: Supplementary file 1 — Additional file 1: Supplementary Table 1. Identified themes for needs analysis and evaluation of a postpartum community program. [file 12884_2022_4913_MOESM1_ESM.docx]

Supplementary Table 1. Identified themes for needs analysis and evaluation of a postpartum community program

| Theme | Subtheme | |  |
| --- | --- | --- | --- |
| Pre-Program | | | |
| Location | | | |
| Participant 7 – ‘I think you might find women are unwilling to travel particularly far’  Participant 9 – ‘if it was just first-time mothers then like an inside place … cause it makes mothers feel regardless of the weather they can get there and not worry about their baby catching a chill or something if it’s outside’  Participant 10 – ‘I reckon you find that people come to one that's local to them.’  Participant 13 - maybe down at the somewhere near a playground or, I have an older child, but I know if it’s anyone with older children that were coming with them, it’s always good to be near a playground. So, you can send the other one off to a playground  Focus Group 1 - ‘And you don’t have to think about, ‘oh, where is it this week?’  Focus Group 1 - ‘Could you have a backup plan at like the URAC at the um, uni or something?’  Focus Group 2 - ‘Yeah, North Wollongong an all yearly surf club.’  Focus Group 2 - ‘You could have wet weather activities, something you could do indoors.’  Health professionals 1 – ‘I mean outdoors just seems nicer because they’re normally desperate to leave rather than be indoors, but you might find anywhere along the bike track where there’s a park or just some free space. Especially if they have other kids, they might have to bring them along if they’ve got toddlers or things like that. It just gives a bit of space to create a friendly environment. And then just being a bit conscious of pram friendly, so wherever there’s plenty of space for paths, parking, those sorts of things.’ | | | |
| Timing | | Day of the week | |
| Participant 5 – ‘Weekends. I’ll be back to work … so Friday-Sunday is good’  Participant 12 – ‘I would probably go a weekday. Because I know weekends, for me, like my husband works during the week so weekends I like to keep free sort of for family time’  Participant 13 - Weekdays definitely weekdays  Participant 14 - Weekdays definitely because I think weekends everybody is with their family  Participant 14 - yeah, I wouldn’t put something on a Monday. I think Tuesday, Wednesday, Thursday usually works the best.  Focus Group 1 - ‘I guess if it runs say Tuesday Thursday if you miss the Tuesday, then you could go to the Thursday’  Focus Group 1 - ‘I think access routine might be a big thing … They might be dropping off other kids to school and then coming to the group, so if it is something close to where they live, it might spark some more interest’  Focus Group 2 - ‘I did ask one lady yesterday, and she said I’m going back after 3 months to work, so she said 6 weeks, so, so some mums are going back to work quick’  Focus Group 2 - ‘Should we keep it away from school holidays? Because all the kids won’t want to’ | | | |
| Timing | | Time of day | |
| Participant 4 – ‘will probably be influenced a lot by sleep/feed schedules’  Participant 7 – ‘I would definitely do mornings as opposed to arvos’  Participant 9 – ‘they might remember that the early newborn stage though is OK 'cause they can just sleep in the pram and sort of the next stage up you need to talk to someone who's got like a like a four-month-old plus 'cause they will be instead of like a pattern at about four months and some babies don't like to, or some moms don't like to break that pattern and they do have a morning sleep’  Participant 10 - Morning. morning. Hands down morning. Afternoons here are best spent at home’  Participant 11 – ‘yeah because the mums group for my second baby were meeting in the afternoon and it was sort of a bit hard. You’re waiting around all day and then I think yeah, I personally find the mornings a bit easier.’  Participant 12 –‘the day can go pear shaped quickly so mornings are always easier’  Participant 13 – ‘I think most people prefer mornings. With babies and kids, you’re sort of happier to unsettle their sleep in the mornings and then at lunchtimes for a decent afternoon sleep. Generally, what most people like to do  Participant 14 - The timing changes as the baby grows. I remember when they were like under 3 months old you could meet at 10-10:30am but now you know that doesn’t work with naps. So once they’re like 6 months or above it needs to be like 11 or 11:30. So the older the baby gets, and then once they go down to one sleep, the time needs to go back to like, you know, 9:00 or 10:00.  Focus Group 1 - ‘10 to 1 or 10 to 12 or something like that. And then they (the babies) would nap, isn’t it?’  Focus Group 2 - ‘Maybe make one in the morning, one in the afternoon.’  Health professionals 1 - ‘So most of the time I find women, if they’re bringing bub to the appointment, it’s that mid-morning time seems to be the most kid and bub friendly. So too early, they’re still organising from the night before potentially not having a good night’s sleep and then they normally try to time it around feeds and like that so depending on what bub’s schedule is. Anything in the afternoon seems to mess with their sleep and evening routine and stuff like that.’ | | | |
| Timing | | Length of program | |
| Participant 7 – ‘If you have it weekly, you’ll have people that won’t come every week, it might be too much’  Participant 8 – ‘yeah I reckon in a block because you then get in a bit of a rhythm’  Participant 9 – ‘but yeah you might start to see them every second week or every week. They might miss one week and then it turns into like a little bit of a support group for them’ | | | |
| Format | | Order | |
| Participant 4 – ‘education first then walk … then can walk and talk about what was taught/learnt/presented/ask questions’  Participant 7 – ‘I think probably the education would be better first’  Participant 7 – ‘I wouldn’t start the education for 15 minutes after it’s meant to start or something and then they [mums] can go if they need to’  Participant 8 – ‘I think education first … you could even talk to the other mums on the walk about stuff you’ve learnt or share experiences and stuff’  Participant 9 - I think maybe chat first because then mothers that need to run off can then go, I'm not gonna go for the walk or you know something happens to baby. babies are so unpredictable like if the babies fussy the mom can then go, I'm just going to I'll miss the walk today I just got home yet and at least they're still getting their educational component of it yeah yeah’  Participant 13 - I think it depends what your trying, like if your focus is more on the education then I’d say do the education at the end because people will likely be late with babies.  Participant 14 - I think education then the walk because I think it would be good to debrief it and talk about stuff | | | |
| Format | | Baby age | |
| Participant 14 – ‘if there were a group of women who started and everyone in the group had a baby between, I don’t know, who was born in a month or two months, then we’d all have similar issues and be moving through at the same time, then I’d be more likely to go and get support from that. Because if I were in a group where a mum had a one-year-old when I had a four week old, our issues would be totally different’  Participant 14 - That for me would be a factor is I’d want to be placed in a group with babies similar age to my baby | | | |
| Topics | | Ideas for mum | |
| Participant 2 – ‘pelvic floor – pelvic floor physio. Returning to exercise … if I was a first-time mum I would also be interested in: breastfeeding support, scales, sleep tips and tricks, food and feeding tips, recipes etc.’  Participant 5 – ‘exercise for preventing diabetes type 2 in the future as well as GDM [gestational diabetes] in the next pregnancy’  Participant 7 – ‘Pelvic floor physio kinda stuff’  Participant 9 – ‘and like yeah good exercises postpartum yeah and its sort of at different stages you know at six weeks what you can do at six weeks is gonna look very different to what you can do at 12 weeks well yeah one year’  Participant 10 – ‘the physio for sure all of that. I mean, I accessed a lot of that independently myself, but like the physio postpartum stuff exercises and like pelvic floor and abdominal separation stuff like it's all all really handy to know or have a refresher on yeah.  Participant 10 – ‘I think the second time around, the hardest part of second time baby is navigating your your first child’s behaviours and how to. How to make it a smooth transition for them too’  Participant 12 – ‘so like women’s health physio’  Participant 12 – ‘maybe like a postpartum psychologist so like an antenatal specialist who could talk through like um mum’s mental health and like I guess things like postpartum depression and anxiety but also like postnatal depletion in general. So a specialist in that kind of area to talk about managing your mental health. What else, women’s health physio, even just like a specialist like a postnatal GP for talking through things like managing hair loss and the more physical sides of things as well. That would be really cool to know like what’s normal, what’s not normal for things like hair loss postpartum’  Participant 12 – Maybe even like a nutritionist to look at diet and like breastfeeding and um managing all of that ‘exactly and ways to boost your calories in a healthy way while breastfeeding instead of just eating chocolate.’  Participant 14 - maybe someone who is an expert on pelvic floor health  Participant 14 - like a relationships, a relationships [psychologist, talk about how your intimate relationship with your partner changes and like talking about normalising that and what you’re going to go through and creating an open space to talk about like sex and intimacy following your baby.  Health professionals 1 – ‘So, I think early postpartum is pretty well covered. So I think you could still cover it in your program of you know potentially having a lactation consultant or something like that … Um but I think then as you go throughout the postnatal period is then you’re probably looking at something more to be sustained for a long period.  Health professionals 1 – ‘So, whether it’s nutrition for mum while she’s breastfeeding so that she has to keep her fluid up and she needs to be eating certain types of food to create appropriate breastfeeding. As bub moves to solids does then her nutrition have to change. Exercise would be a big one you could add as a component. Most women are desperate to exercise but are time poor so if you could have that within a set like mother’s group scenario then they’re going to get a lot more out of it I think.’ | | | |
| Topics | | Ideas for Baby | |
| Participant 7 – ‘it’d be like feeding, sleeping, settling, and breastfeeding. Bottle and boob. Oh like tummy time and that sort of stuff, [baby] development’  Participant 7 – ‘if you could do something like a GP or something on childhood illnesses or rashes and things to look out for. Like when to go to the emergency department or when to you know call one of the health lines’  Participant 8 – ‘sleep would definitely be one … it’s been our main topic that we’d had to research and learn’  Participant 11 –‘ I feel like a paediatrician or a doctor would be handy. Because I guess looking at when you should take your baby to get checked if there’s something yeah’  Participant 12 – ‘I’d reckon sleep consultants and maybe even like paediatric dietitians or um lieks omeone who is kind of a specialist with explaining different ways of introducing foods like purees or baby-led weaning and stuff like that’  Participant 13 – ‘But I would be very interested in talking with a psychologist regards to my older child and how he’s coping with the transition of having a sibling.  Participant 14 - then the big topics we’re all obsessed with in our mothers’ group are like sleep, so having a sleep consultant who came and spoke about the fundamentals of sleep and gave some ideas about how to do good sleep, good routine, a little bit about sleep training, but for women who don’t believe in sleep training, a little bit more different approaches to sleep. Like we all talk about that every single day. So to have access and they’re really expensive to hire a sleep consultant.  Participant 14 - And also, someone who came, maybe like a baby nutritionist, who had little recipes and ways you can inject nutrition and easy meals for your baby. But also, what would be really helpful like a first aid would be amazing. Baby first aid. If they came and gave some advice on choking, you know rashes, which ones are important, which ones to look out for, what are the signs of the cold or the flu | | | |
| Activities or Topics | | Barriers to Attendance | |
| Focus Group 1 - ‘You’re not going to be walking on a rainy day.’  Focus Group 1 - ‘If she’s 20 minutes late and I’ve missed the education, then … what’s the point of showing up… I think that would be the hardest thing’  Focus Group 1 - ‘The majority of women they’re still working, they’ve got other little kids and… they go to antenatal clinic, it’s just so many appointments’  Focus Group 2 - ‘wonder if we could get… a fenced off area if someone watches them [their children]… and there are even parks that have fences around them’  Focus Group 2 - ‘I think that the biggest barrier will be… getting the women we want to turn up, rather than the women that are already probably quite educated’  Health professionals 1 – ‘Women are time poor um I think if you make the transition quite easy for them to attend, which again you’d have to get some feedback from participants, of what makes that run better. But I think once they’re committed, they’ll stay committed, it’s just getting them to take that first leap.’  Health professionals 1 – ‘But definitely being aware of women who have other kids. So, if it’s their first, I normally find they’ve only got them or the bub and bub’s always with them so it’s easier for them to get out and move. When you’ve then got a toddler to negotiate around, that can be a bit more tricky so whether you emphasise that it’s kid friendly, you can bring the toddlers, have a space for them. Whether that’s a little creche or something um so that’s a barrier that’s taken away hopefully for them.’ | | | |
| Program Evaluation | | | |
| Social Media | | How they heard about the program | |
| Social Media use  Participant 16 – “I’m pretty sure it was through social media”  Participant 17 – “I think it was an ad of Facebook”  Participant 19 – “I remember, it was one of the girls in mother’s group. She posted the link… it’s actually really good, it’s on now… jump on if you can” | | | |
| Social Media | | Facebook group | |
| Participant 16 – “I think you were trying to get the participants to kind of be more interactive on the Facebook group, but I felt that that wasn’t really happening”  Participant 21 – “I think probably the thing that would’ve been good or helpful would be the Facebook group that you sort of started and nobody really seemed to engage in… I thought that was a really good idea” | | | |
| Social Media | | Recordings being posted on the group | |
| Participant 16 – “I didn’t feel it was easy [to navigate] or maybe there was quite a big delay”  Participant 16 – “through Facebook that’s perfectly fine”  Participant 20 – “If I knew about the recorded options, I definitely would have watched those”  Participant 22 – “because of the Facebook group … I went back and watched all the videos”  Participant 22 – “it was fine to just scroll back down” | | | |
| Activities or topics | | Feedback regarding presented topics | |
| Participant 16 – “women’s health physio I thought was really helpful”  Participant 16 – “if I could only tune in for one portion … it was more information … I wasn’t interested in the exercise portion”  Participant 18 – “It was more irrelevant to me … my little guy in October was already past that six months and being a second time mum”  Participant 19 – “it was a personal trainer and I thought hell no, there’s no way I’m doing that shit anytime soon”  Participant 21 – “I vividly remember the session on breastfeeding … yeah I found the breastfeeding one fascinating”  Participant 22 – “I was drawn to the working mums one but also the physio one”  Participant 22 – “I thought the concept of this program was sensational, but as a second time mum of a 6 month old when the program started I found a lot of the things irrelevant. I’d defiantly recommend to a first time mum of a new born” | | | |
| Activities or topics | | Ideas for further topics | |
| Participant 15 – “recommend speech pathologist for eating and … mental health professionals”  Participant 16 – “[talking about a different program] I think we had a child psychologist or someone talking about language development … you know it’s interesting but it wasn’t really applicable to me at that point”  Participant 18 – “the general planning to prepare for work or going back to work”  Participant 21 – “one on sleep but that was a big think for me postpartum and figuring out kids sleep” | | | |
| Activities or topics | | Barriers to attendance | |
| Participant 15 – “remembering was a problem for me”  Participant 15 – “if it didn’t fit in with their schedule then I just wouldn’t do it”  Participant 19 – “I just couldn’t hear it over the screaming”  Participant 23 – “I don’t think I attended because Melbourne was finally being released from a 4-month lockdown and sitting behind a screen watching a webinar was the last thing I ever felt like doing” | | | |
| Recommendations | | Would you recommend the program | |
| Participant 15 – “yes I would [recommend to a friend]”  Participant 18 – “I think the program is definitely worthwhile for … a first-time mum”  Participant 18 – “could definitely see the value of it though if I was just starting fresh”  Participant 19 – “Yeah I definitely would”  Participant 22 – “yes [I would recommend the program to a friend]”  Participant 22 – “yeah, I definitely recommend it to a friend” | | | |
| Structure | | Ratio of Education to Physical activity | |
| Participant 18 – “yeah the content delivery was a non-issue”  Participant 20 – “maybe some visuals, sometimes it’s really nice to be able to watch a recorded person talk”  Participant 22 – “for some days … I’d come in for the speakers just to jump into the physical part, but I think dividing it sort of into two makes it interesting and interactive” | | | |
| Structure | | Questions and answers | |
| Participant 18 – “I think potential to like send or submit pre-questions around that topic”  Participant 19 – “have the questions like people can write in questions throughout”  Participant 20 – “Like a Q&A is always great as well”  Participant 21 – “I did like the questions and answers | | | |
| Timing | | Program time | |
| Participant 15 – “I was free unless bub was you know really being fussy or something”  Participant 21 – “I found the timing on some days was quite hard with when the kids needed to go to sleep”  Participant 21 – “I don’t know if this is a solution that you’ve thought of is to not having exactly the same time for every session”  Participant 21 – “evening times are normally for people to wind down”  Participant 22 – “yeah 9:30am or 10am but yeah 9:30am would be fine”  Participant 24 – “I found the morning time slot hard to attend with two others young child (though one was at school) but the opportunity to watch the recording later was great” | | | |
| Platform | | Current format | |
| Participant 16 – “I like it when it’s a live webinar format”  Participant 19 – “I think the webinar was best way because of covid”  Participant 21 – “I did find it hard that they were online because I would have liked the excuse to go to something if that makes sense”  Participant 21 – “I mean if the video aspect of it wasn’t there I probably wouldn’t have been as interested”  Participant 22 – “it was just so good to do it from home as well” | | | |
| Platform | | Potential platforms for future programs | |
| Participant 15 – “Audio is definitely easier for me”  Participant 17 – “my husband thought it would be useful if it could be like I audio form … he doesn’t really have the time to sit down and watch videos”  Participant 21 – “I would have … loved something like that to have been in person, to have like met other mums in that situation”  Participant 22 – “obviously, things in person would be good but I think that’s kind of, that platform, any kind of webinar is really good because of the times we live in now” | | | |
